# Analyzing public discourse of dementia from Spanish and English tweets: a comparative analysis with other neurological disorders

**DOI:** 10.3389/fneur.2024.1459578

**Published:** 2024-10-24

**Authors:** Javier Domingo-Espiñeira, Óscar Fraile-Martínez, Cielo García Montero, Francisco Jesus Lara Abelenda, Jesús Porta-Etessam, Laura Baras Pastor, Leticia I. Muñoz-Manchado, María Arrieta, Mahdieh Saeidi, Miguel A. Ortega, Melchor Alvarez De Mon, Miguel Angel Alvarez-Mon

**Affiliations:** ^1^Department of Medicine and Medical Specialities, Faculty of Medicine and Health Sciences, University of Alcala, Alcala de Henares, Spain; ^2^Ramón y Cajal Institute of Sanitary Research (IRYCIS), Madrid, Spain; ^3^Departamento Teoria de la Señal y Comunicaciones y Sistemas Telemáticos y Computación, Escuela Tecnica Superior de Ingenieria de Telecomunicación, Universidad Rey Juan Carlos, Fuenlabrada, Spain; ^4^Unidad de Cefaleas, Servicio de Neurología, Instituto de Investigación Sanitaria San Carlos, Hospital Clínico San Carlos, Madrid, Spain; ^5^Departamento de Medicina, Facultad de Medicina, Universidad Complutense de Madrid, Madrid, Spain; ^6^Clínica OPbaras, Psiquiatría y Psicología, Sevilla, Spain; ^7^UGC North of Cadiz, Mental Health Inpatient Unit, General Hospital, Jerez de la Frontera, Spain; ^8^Serious Mental Disorder Research Group, Cadiz Biomedical Research and Innovation Institute, Cádiz, Spain; ^9^Servicio de Psiquiatría, Hospital General Universitario Gregorio Marañón, Madrid, Spain; ^10^Columbia University Medical Center, New York State Psychiatric Institute, New York, NY, United States; ^11^Service of Internal Medicine and Immune System Diseases-Rheumatology, University Hospital Príncipe de Asturias, Centro de Investigación Biomédica en Red de Enfermedades Hepáticas y Digestivas (CIBEREHD), Alcala de Henares, Spain; ^12^CIBERSAM-ISCIII (Biomedical Research Networking Centre in Mental Health), Madrid, Spain; ^13^Department of Psychiatry and Mental Health, Hospital Universitario Infanta Leonor, Madrid, Spain

**Keywords:** dementia, X (Twitter), neurological disorders, artificial intelligence, machine learning, social perceptions

## Abstract

**Introduction:**

Dementia comprise a broad spectrum of cognitive declines affecting 47 million people worldwide, with numbers projected to reach 131 million by 2050. Predominantly associated with older adults, dementia can also impact younger individuals, having a significant impact on daily functioning of the affected patients, relatives, caregivers and the socioeconomic system. Recent research underscores the utility of social media, particularly X (previously designed as Twitter), in understanding public perceptions and sentiments related to neurological disorders. Despite some initial studies have explored social perceptions of dementia in X, broader and deeper analysis of this condition is still warranted.

**Materials and methods:**

In this retrospective study, we collected and examined all tweets posted in English or Spanish from 2007 to 2023 that mentioned dementia and compare the information with other highly representative neurological disorders like migraines, epilepsy, multiple sclerosis, spinal cord injury, or Parkinson's disease. We developed a codebook to analyze tweets, classifying them by themes such as trivialization, treatment perceptions, and etiopathogenesis. Manually categorized tweets trained machine learning models, BERTWEET for English and BETO for Spanish, which then classified larger datasets with high accuracy. Statistical analysis, including ANOVA, Kruskal-Wallis, and chi-square tests, was conducted to explore linguistic and cultural differences in perceptions of neurological disorders, with results visualized.

**Results:**

Our study reveals that dementia is by far the most frequently discussed neurological disorder on X. Likewise, this condition appears to be the most trivialized neurological disorder in Spanish tweets and the second most trivialized in English tweets, with notable differences in geolocation data. Additionally, we found significant differences in perceptions of dementia treatment and associated sentiments between Spanish and English tweets. Furthermore, our study identified varying perceptions of medical content (etiology) and non-medical content (positive/negative experiences and aid requests) related to dementia and other neurological disorders, unveiling a complex landscape of these topics on X.

**Conclusions:**

This study explores the importance of X as a social platform for addressing various critical issues related to dementia, comparing it with other neurological disorders in English and Spanish tweets. Future research could further investigate the valuable role of social media in understanding public perceptions and needs regarding dementia and neurological disorders among X users.

## 1 Introduction

Dementia encompasses a range of cognitive declines impacting daily functioning, representing a syndrome with diverse causes spanning neurological, neuropsychiatric, and medical conditions. Globally, 47 million people live with dementia and, by 2050, the number is expected to increase up to 131 million. (1). Dementia is commonly related to elder people; however, it is important to remark that dementias can also affect younger individuals (2). Alzheimer's disease is the most common type of dementia, followed by vascular and Lewy body dementias, although they are not the only causes of dementia (3). The Diagnostic and Statistical Manual of Mental Disorders, Fifth Edition, (DSM-5) now encompass dementia under the term of Major Neurocognitive Disorder (MND) (4). Dementia or MND is a progressive condition, with gradually worsening symptoms. Because of this, it has a great impact on both people who suffer from this condition as well as their relatives and caregivers, also being a major cause of social stigma (5). Likewise, the economic burden of dementia globally was estimated to be US $1313.4 in 2019 (6) whereas a significant increase impact is projected to occur in the next decades (7). Therefore, the growing impact of dementia and associated problems for the affected individuals, their environment and the society are supported by a consistent body of evidence. The analysis of public perceptions and concerns related to dementia represents an important field of research, allowing to better understand the consequences of this condition on the affected individuals and the entire society.

Social media represents an excellent platform to understand public perceptions related to various topics. Indeed, the data show that 58% of the global population actively participate in social media, providing researchers with valuable insights into health factors via shared lifestyles, behaviors, perceptions and experiences (8). In this context, previous works have found that X represents a useful tool to explore public views on different diseases, including neurological disorders (9–11). Advantages from using this platform include that this social network is often seen as a secure and impartial space where individuals feel comfortable sharing candid experiences, even on delicate subjects (12). In the event of dementia, previous literature has found some interesting results extracted from analyzed data in X. For instance, the studies highlight that social stigma and mental health advocacy are common topics of people tweeting about dementia, evidencing the relevance of both elements in public discourse (13, 14). Also, other works state that X is a great medium to disseminate health and research information related to dementia, as well as to explore perceptions of people affected by dementia, as well as their families and caregivers (15, 16). Likewise, studies in the Twittersphere have been conducted in recent years to evaluate public perceptions and sentiments associated to dementias during and after the coronavirus disease 19 (COVID-19) pandemic (17–19). Thus, the relevance from studying dementias on X is supported by a growing body of evidence; however, broader, and deeper information found in this social platform is required for a better understanding of social perception of this condition.

Past works have remarked the relevance from including Tweets from different languages to describe possible cultural or regional differences related to certain topics (20–22). Also, the inclusion of artificial intelligence and machine learning approaches, along with sentiment analysis and geolocation of tweets also provide valuable insight into the significance of public perceptions (23). Similarly, the comparison of multiple topics and perspectives across different types of neurological disorders has also been considered in past works (24), having the potential to face the perceptions around distinct disease entities. In this context, the aim of the present study is to compare Spanish and English Tweets discussing dementias facing this condition with other common neurological disorders such as migraines/headaches, epilepsy, multiple sclerosis (MS), spinal cord injury (SCI) and Parkinson's disease (PD). We have decided to focus on these entities while excluding other representative neurological disorders like stroke due to their distinct chronic and progressive nature. Stroke is primarily an acute condition requiring immediate medical intervention, which contrasts with the long-term management needed for the selected disorders. Chronic neurological diseases generate sustained public interest and healthcare challenges, making them more suitable for infodemiological analysis. By narrowing our scope, we aim to provide a clearer understanding of information-seeking behaviors and public health impacts associated with long-term neurological conditions.

## 2 Materials and methods

### 2.1 Collection of X data and content analysis process

In this retrospective study, we collected and analyzed all tweets posted in English or Spanish between 2007 and 2023, referring to headache disorders, dementia, epilepsy, MS, SCI or PD using a search engine called Tweet Binder that has access to 100% of publicly available tweets. Thus, we collected all tweets containing any of the following keywords in English or in Spanish: headache, dementia, epilepsy, multiple sclerosis, spinal cord injury, or Parkinson. Our study timeframe spans from X's foundation in 2007 to the beginning of our study in 2023, enabling a comprehensive exploration and analysis of public opinion over an extended period. In addition to the content of the tweets themselves, we also collected additional information such as the date it was posted, the number of retweets, or the number of likes and user description. The number of retweets and likes served as indicators of user engagement and interest in the tweeted content.

### 2.2 Content analysis process

We created a codebook based on our research questions, our previous experience in analyzing tweets, and what we determined to be the most common themes. In each tweet, we analyzed whether the reference to the disease was serious or trivializing, and whether the disease was considered curable or incurable. In cases where the disease was considered curable, we distinguished between tweets stating that the disease was curable with professional help and those stating that it was self-manageable. Next, we analyzed the etiopathogenesis of the disease. Specifically, we distinguished between tweets that considered the disease to be due to: (1) Genetic or biological causes; (2) COVID-19; (3) Environmental stressors; (4) Multifactorial causes; (5) Psychological stress; (6) Weakness or inability to cope with stressors. Finally, we analyzed the non-medical content, distinguishing between tweets that referred to personal experiences with the disease (as a patient or family member) and those that were requests for assistance or promotion of prevention programs.

### 2.3 Machine learning application

With the development of new technologies, the use of artificial intelligence and machine learning has expanded across diverse fields, with its implementation in social listening emerging as particularly widespread. Machine learning is a field of artificial intelligence where computer systems learn from data to emulate the human reasoning and perform task in a larger scale (25). Therefore, machine learning is crucial in assessing extensive datasets that would be impractical to evaluate manually. Machine learning comprises three primary types: supervised, unsupervised, and semi-supervised. In this study, we employ semi-supervised learning, blending aspects of both supervised and unsupervised methodologies by leveraging both labeled and unlabelled data. The methodology employed extends beyond the conventional manual analysis. The goal is to develop a model that emulates the analyses of an expert, classifying a large number of tweets that would be impossible for a human. Toward this end, a professional underwent a manual classification of 1,000 English tweets and 1000 Spanish tweets in several categories to train two machine learning algorithms.

Before the machine learning application, the tweets underwent a preprocessing, involving normalization through the removal of special characters, splitting negative contractions, and removing repeated word. Subsequently, the two manually classified databases (Spanish and English) were randomly partitioned into training subset composed by the 75% of the samples ana a test subset composed by the rest 25% of the samples. Finally, a machine learning model was trained for each one of the two datasets using the two train subsets. For the English dataset a transformer-based model known as BERTWEET (26) was used. BERTWEET is a model based on BERT trained with 80 GB of text containing over 860 million English tweets. The choice of this model is based on its widespread use in the literature (27, 28) and its specific training with short texts, similar to the ones we will evaluate Given that BERTWEET is trained in a self-supervised way specifically on English tweets, for the Spanish dataset, we employed BETO (29). BETO is a BERT model trained on a self-supervised way on a Spanish corpus. The process of retrain a previously trained model is called fine-tuning. This process involves adjusting the parameters of a pre-trained model using task-specific data. The objective is to harness the broad knowledge acquired by the model during its pre-training phase on extensive, unlabelled datasets and use it to address more specialized tasks. To check the performance of the model we use the test set, where both models achieved a F1-score higher than 0.7 in all the categories. This methodology ensures the correct classification of the tweets using the machine learning models. Finally, we used the BERTWEET finetuned to classify the remaining 236.978 English tweets. On the other hand, the BETO finetuned model classified the Spanish dataset, composed by 101.165 tweets.

Lastly, emotion detection was performed using a model from Hugging Face's machine learning platform called “Emotion English DistilRoBERTa-base” (30) for the English dataset and the model called “robertuito” from the pysentimiento library (31). These models are renowned for their efficacy in detecting Ekman's six basic emotions: anger, disgust, fear, joy, sadness, and surprise, along with the inclusion of the emotion called other (32). The English model achieves an accuracy of 66% on detecting the emotions, surpassing the random-chance baseline of 14% (1/7). On the other hand, “robertuito” achieves 70% accuracy for the Spanish corpus.

### 2.4 Statistical analysis

The statistical analysis of the data was conducted utilizing both parametric and non-parametric tests to evaluate differences between groups in categories associated with mental illnesses on social media by language or continent. Initially, data integrity was verified, confirming the absence of missing data in the relevant variables. Tests were conducted to verify the necessary statistical assumptions for ANOVA application. These tests included: (a) Shapiro-Wilk test to assess the normality of residuals; (b) Levene's test for homogeneity of variances among groups. Since the necessary assumptions for performing ANOVA were not fully met, alternative methods were chosen. A simplified ANOVA was conducted to confirm the aforementioned. Simultaneously, the non-parametric Kruskal-Wallis test was applied. The results were subsequently adjusted using the Benjamini-Hochberg and Holm methods to control the false discovery rate and the Type I error rate, respectively. To further explore differences between specific groups within categories, for each disease and language or continent, the Dunn Test was employed. This *post-hoc* test allowed for the comparison of group pairs within each category.

This combined methodology of parametric and non-parametric analysis, along with adjustments for multiple comparisons, provided a comprehensive assessment of statistically significant differences in the usage of terms associated with mental illnesses in different linguistic and cultural contexts. Additionally, the chi-square (χ^2^) test was used to determine if the observed differences between the frequencies of categories associated with different diseases, by language or continent, and the expected frequencies were statistically significant. Furthermore, descriptive analysis was presented both in tables and various forms of graphical representation, such as radar charts for emotional reactions, line graphs for temporal evolution, geographic heatmaps for the distribution and density of categories by disease and country, and clustered bar graphs for the comparison of different categories for each disease by language or continent. These analyses were conducted using the Python programming language.

## 3 Results

### 3.1 Dementia is the first neurological disorder commented in X for English and Spanish tweets, with significant variations on its temporal evolution and other neurological disorders across languages

**Figure 1:**
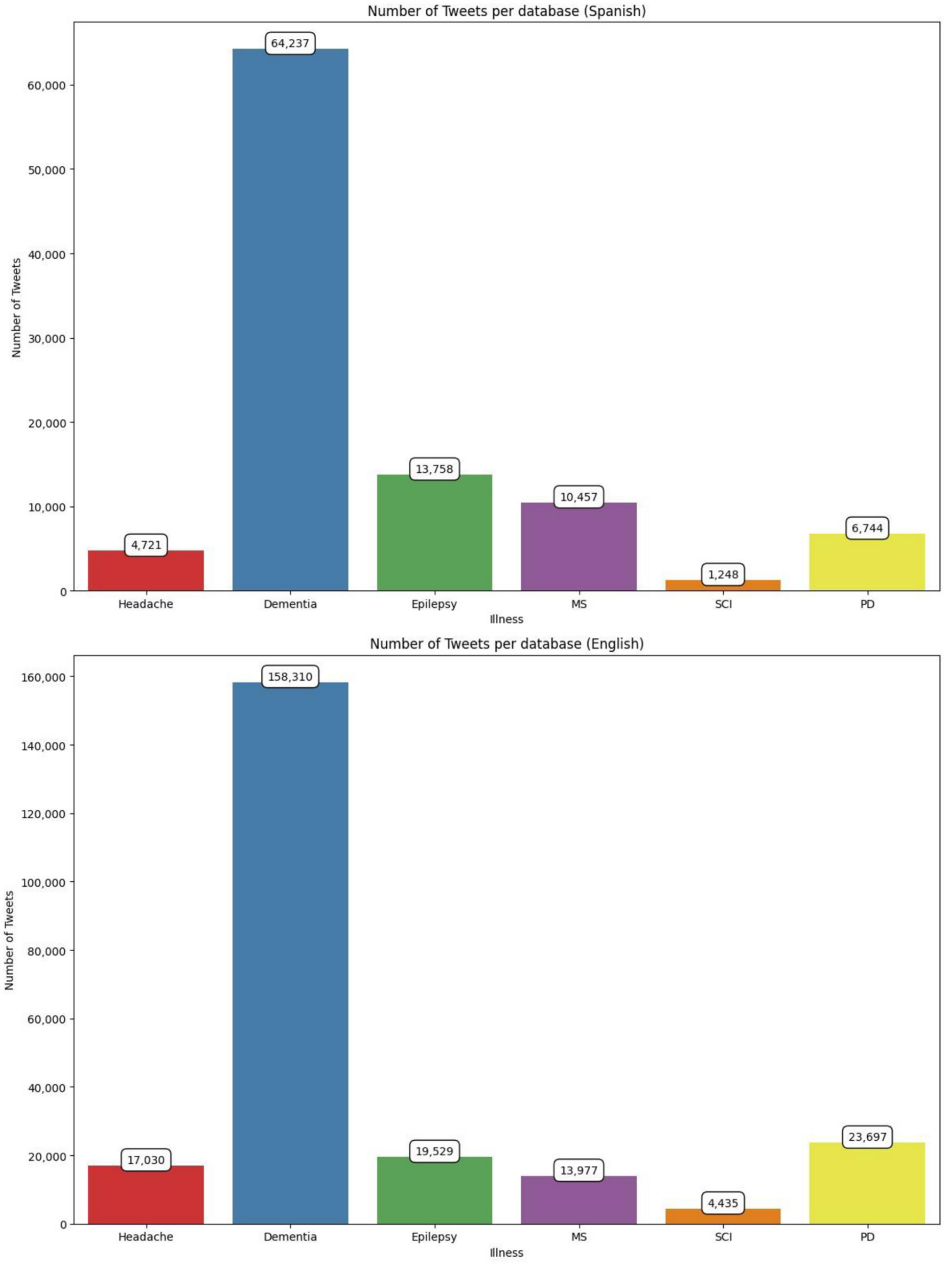
Number of tweets including dementia and other neurological disorders. MS, Multiple sclerosis; SCI, spinal cord injury; PD, Parkinson's disease.

Firstly, we aimed to evaluate the number of Tweets discussing dementia and the remaining neurological disorders included in our study. As observed in Figure 1, dementia was, by far for both Spanish and English tweets, the most commented neurological disorders with 64,237 and 158,310 tweets, respectively. Epilepsy and MS completed the ranking in Spanish tweets, with 13,578 and 10,457, respectively, whereas PD and epilepsy were the second and third most commented neurological disorders in English with 23,697 and 19,529 tweets. SCI was the less commented neurological disorders for both languages (Figure 1).

**Figure 2:**
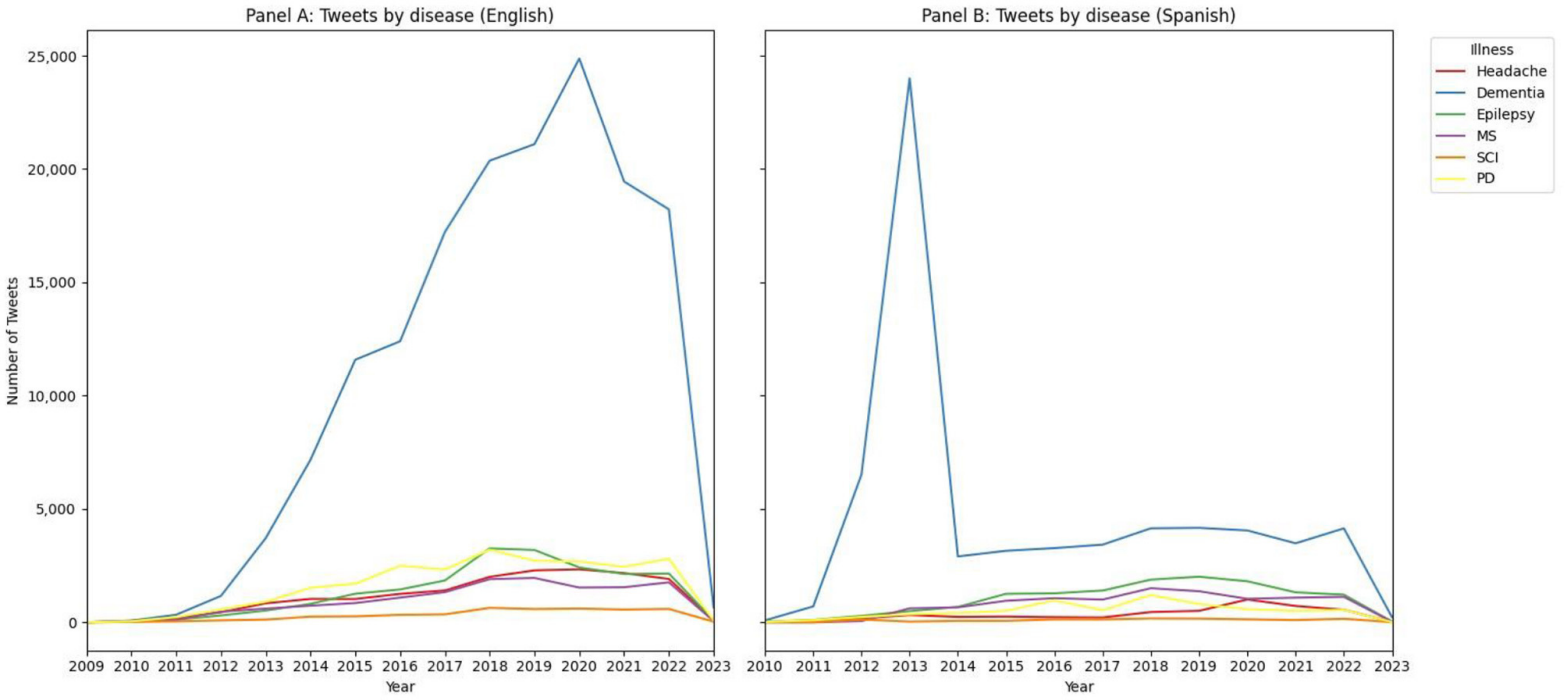
Temporal distribution of tweets including dementia and other neurological disorders. MS, Multiple sclerosis; SCI, spinal cord injury; PD, Parkinson's disease.

Then we assessed temporal evolution of English and Spanish tweets discussing dementia and the other neurological disorders (Figure 2). Regarding temporal evolution of English tweets, we observed a gradual increase of tweets discussing dementia, with a maximum peak reported in 2020 (24,874 tweets) and a progressive decline in 2021 and 2022. We can observe in parallel different patterns for other neurological disorders, being PD and epilepsy the two entities more commonly tweeted after dementia, whereas SCI notably remains as the less commented neurological disorder in our study.

For Spanish tweets, our results evidence a different temporal pattern for dementia, with a maximum peak in 2013 (24,004 tweets in this year) and a drastic decrease in 2014 (2,902 tweets), accompanied by a slight increase in the following years. Epilepsy was the second most commented disorder since 2014 and MS the third.

### 3.2 Dementia was the first neurological disorder trivialized in Spanish and the second in English tweets

**Figure 3:**
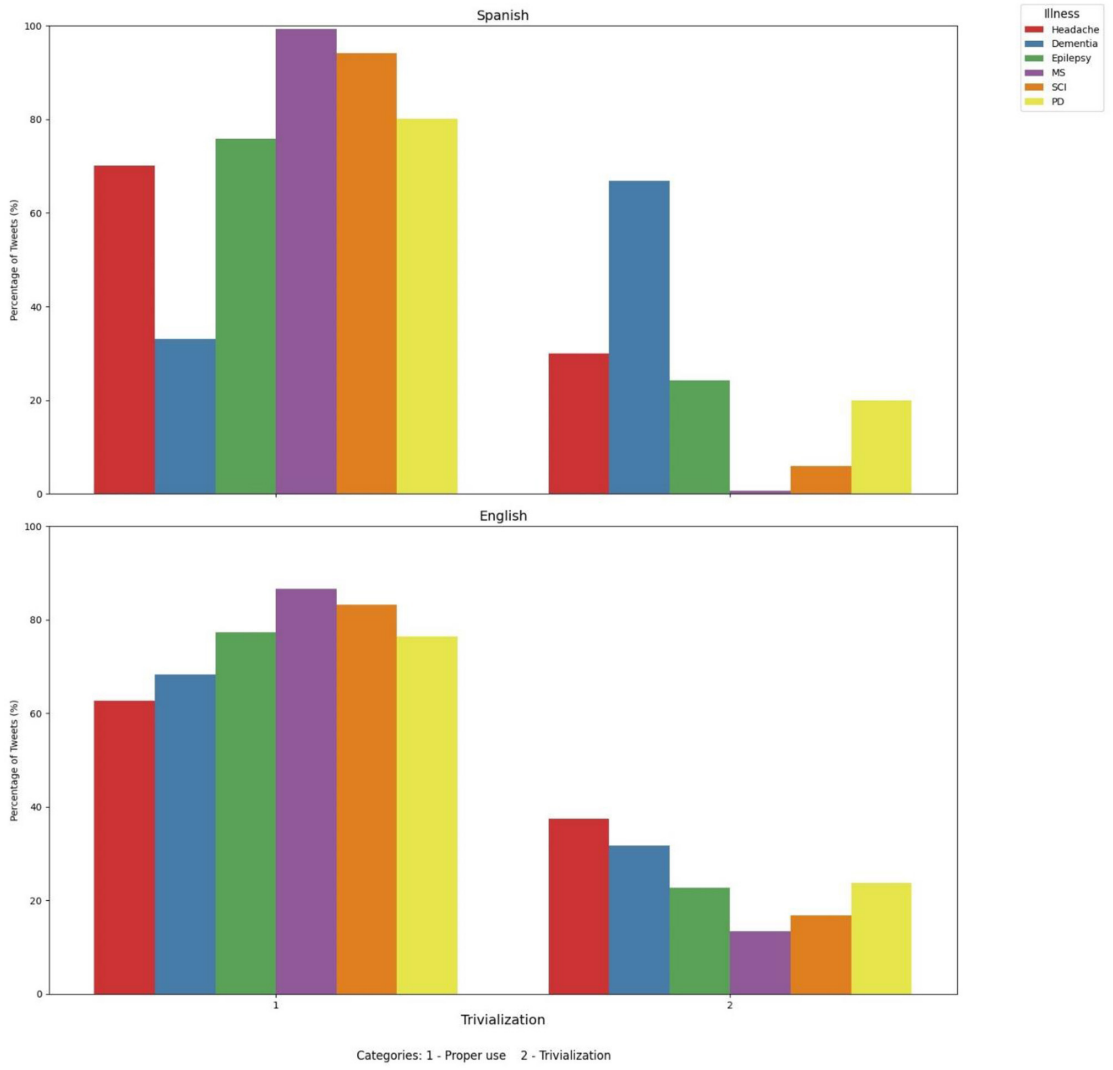
Proper use and trivialization of tweets including dementia and other neurological disorders. MS, Multiple sclerosis; SCI, spinal cord injury; PD, Parkinson's disease.

We then evaluated trivialization of dementia in English and Spanish tweets (Figure 3). Our results show that dementia was the second neurological disorder for English tweets in which we observed trivialization (31.67% of the analyzed tweets), just after headache (37.37%). PD ranked in the third position (23.67%). For Spanish tweets, dementia was by far the most common neurological disorder trivialized (66.9% of tweets discussing dementia), and the second place was for headache (29.97%), closely followed by epilepsy (24.18%).

**Figure 4:**
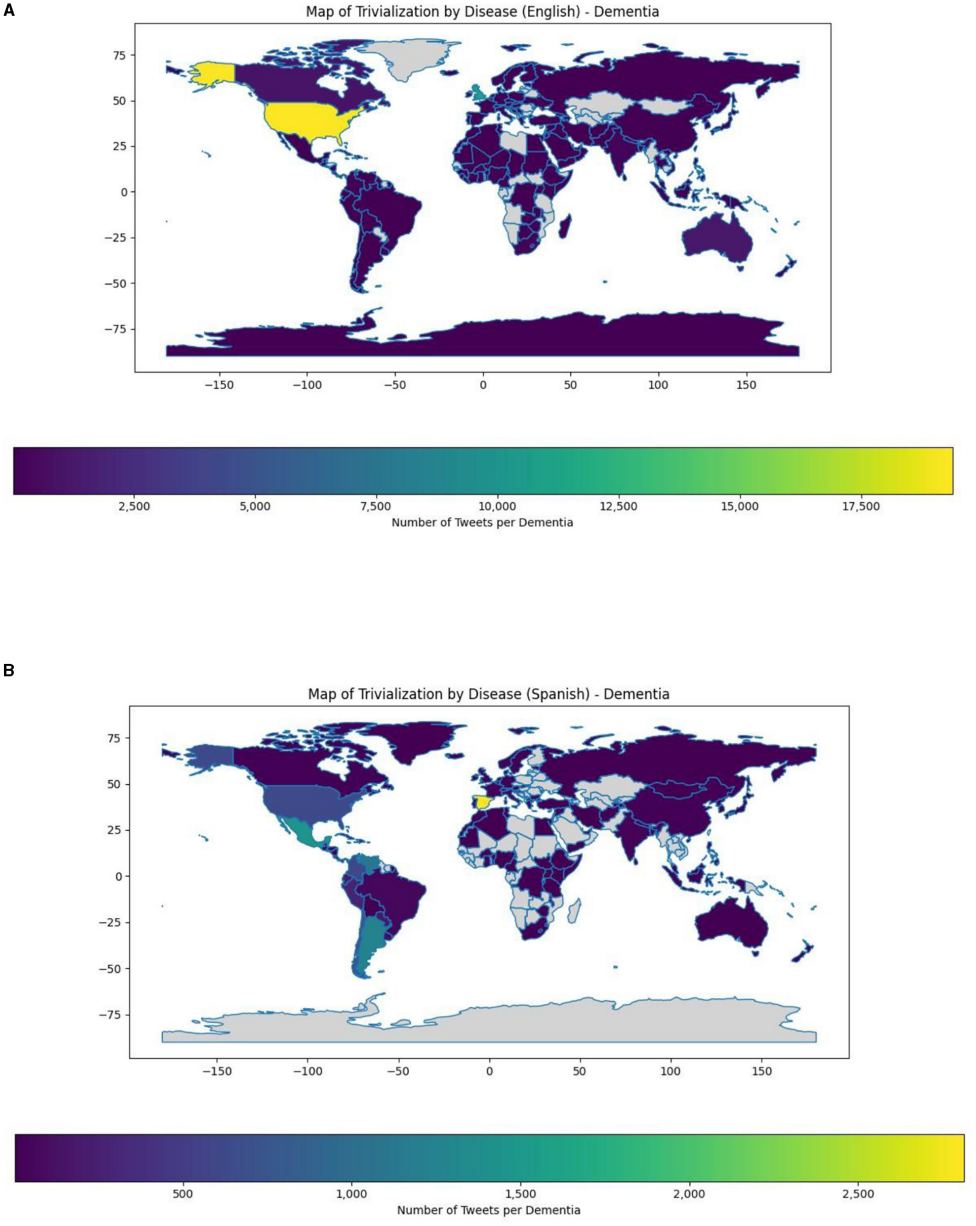
**(A)** Map of trivialization for dementia in English Tweets. **(B)** Map of trivialization of dementia in Spanish tweets.

To deepen on these results, a geolocation map of tweets trivializing dementia and other neurological disorders was also performed. We can observe that for English a broadest number of tweets trivializing dementia can be found in the United States in comparison to other English-Speaking countries like United Kingdom (Figure 4A). For Spanish tweets, we can observe that the broadest number of tweets trivializing dementia can be found in Spain, followed to a lesser extent by countries of Hispanic America like Mexico, Argentina and Venezuela (Figure 4B).

### 3.3 English and Spanish tweets show divergent views and sentiments related to dementia treatment

**Figure 5:**
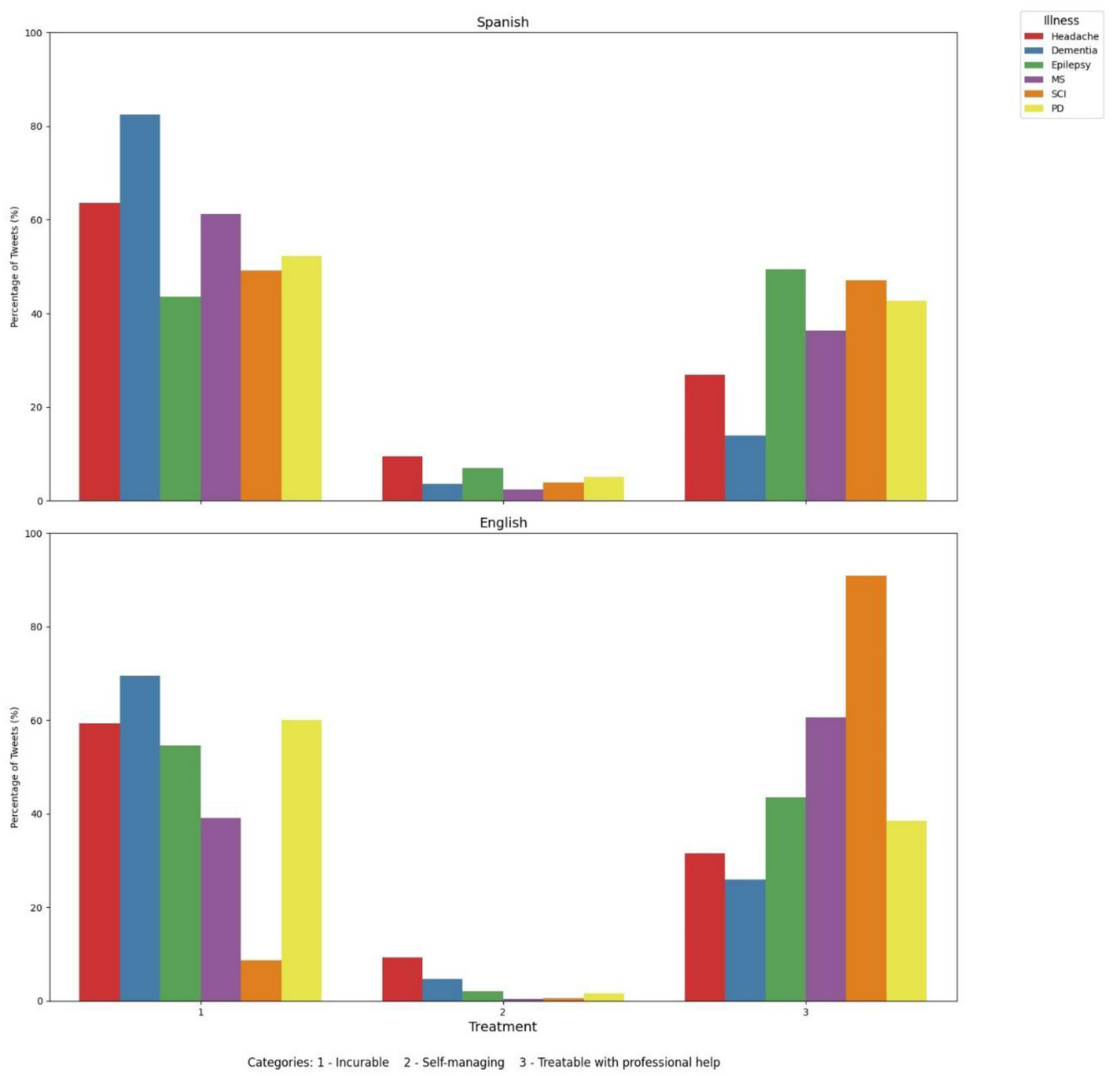
Social perception around treatment of dementia and other neurological disorders in English and Spanish tweets. MS, Multiple sclerosis; SCI, spinal cord injury; PD, Parkinson's disease.

We then evaluate social perceptions around treatment of dementia and other neurological disorders (Figure 5). Our results show that most English tweets perceive dementia as an incurable disease (69.39%), whereas a significant proportion of tweets perceive this condition as treatable with professional help (25.97%) and self-manageable (4.64%). Headache and epilepsy were the second and third neurological disorder more commonly perceived as non-curable (59.22% and 54.48% respectively), whereas SCI and epilepsy were the most common disorders perceived as treatable with professional help (90.82% and 43.43% respectively).

For Spanish Tweets we observed that dementia was by far a neurological disorder perceived as non-curable (82.54% of the Tweets), followed by headache (63.67%) and MS (61.21%). Only a small subset of tweets considered dementia as treatable with professional help (13.89%) and self-manageable (3.57%). 26.84% of tweets and 36.38% perceived headache and MS, respectively, as treatable with professional help.

**Figure 6:**
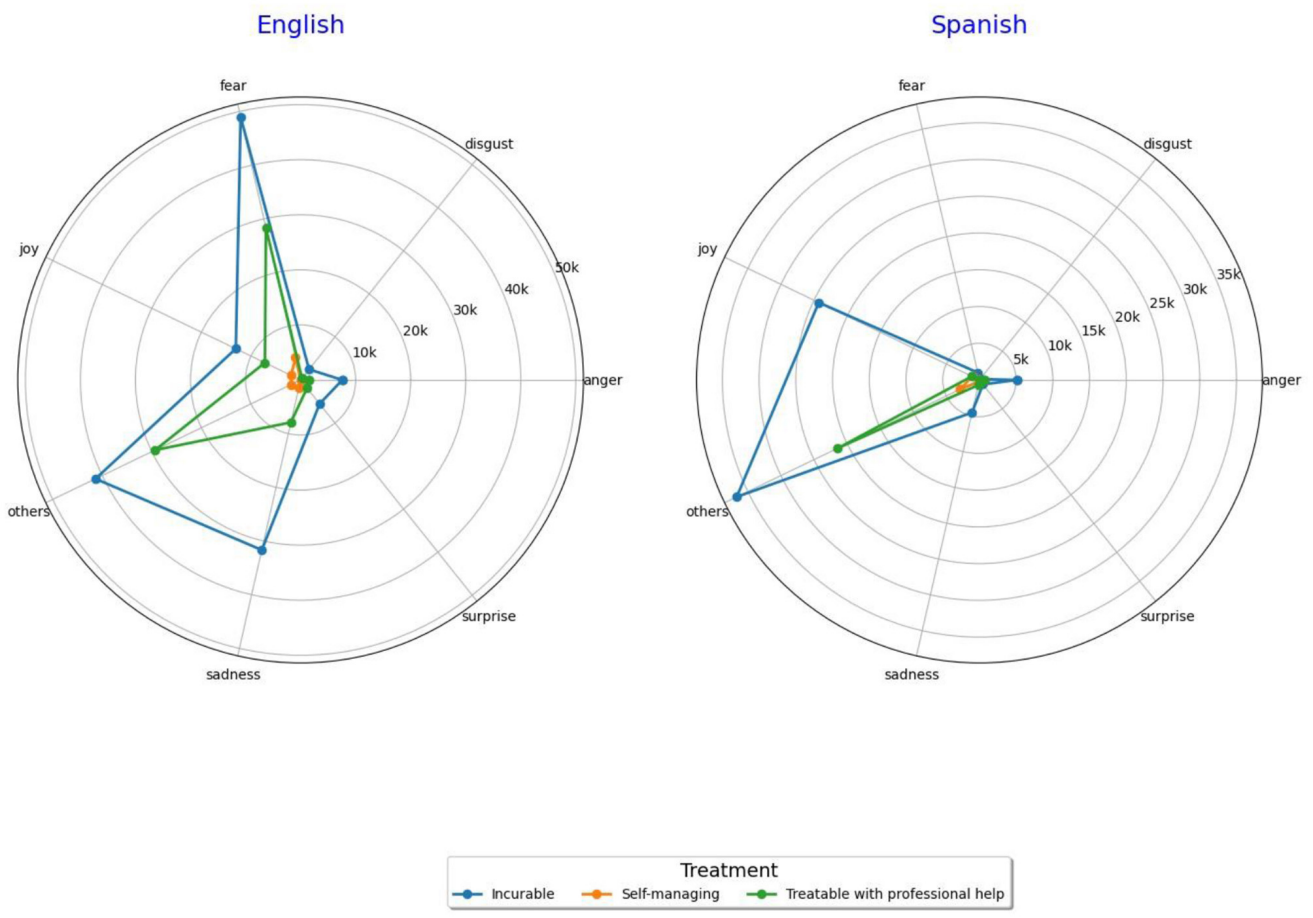
Sentiment analysis regarding treatment conceptions of dementia and neurological disorders in X.

Regarding sentiment analysis (Figure 6) we observed that for English tweets the perception of neurological disorders like dementia as non-curable are associated with feelings of fear, sadness and others, whereas joy, anger and surprise can also be detected to a lesser extent. Conception of neurological disorders as manageable with professional help is more related to fear and other sentiments, with joy and sadness also detected.

For Spanish tweets we observed that conception of neurological disorders as non-curable is associated more clearly with other sentiments and joy, with few of them showing anger and sadness. Little representation can be noticed of fear. For the perception of neurological disorders as treatable with professional help, other sentiments different than the exposed in the graphic are predominant.

### 3.4 English and Spanish tweets have a different discourse around the etiological mechanisms of dementia

**Figure 7:**
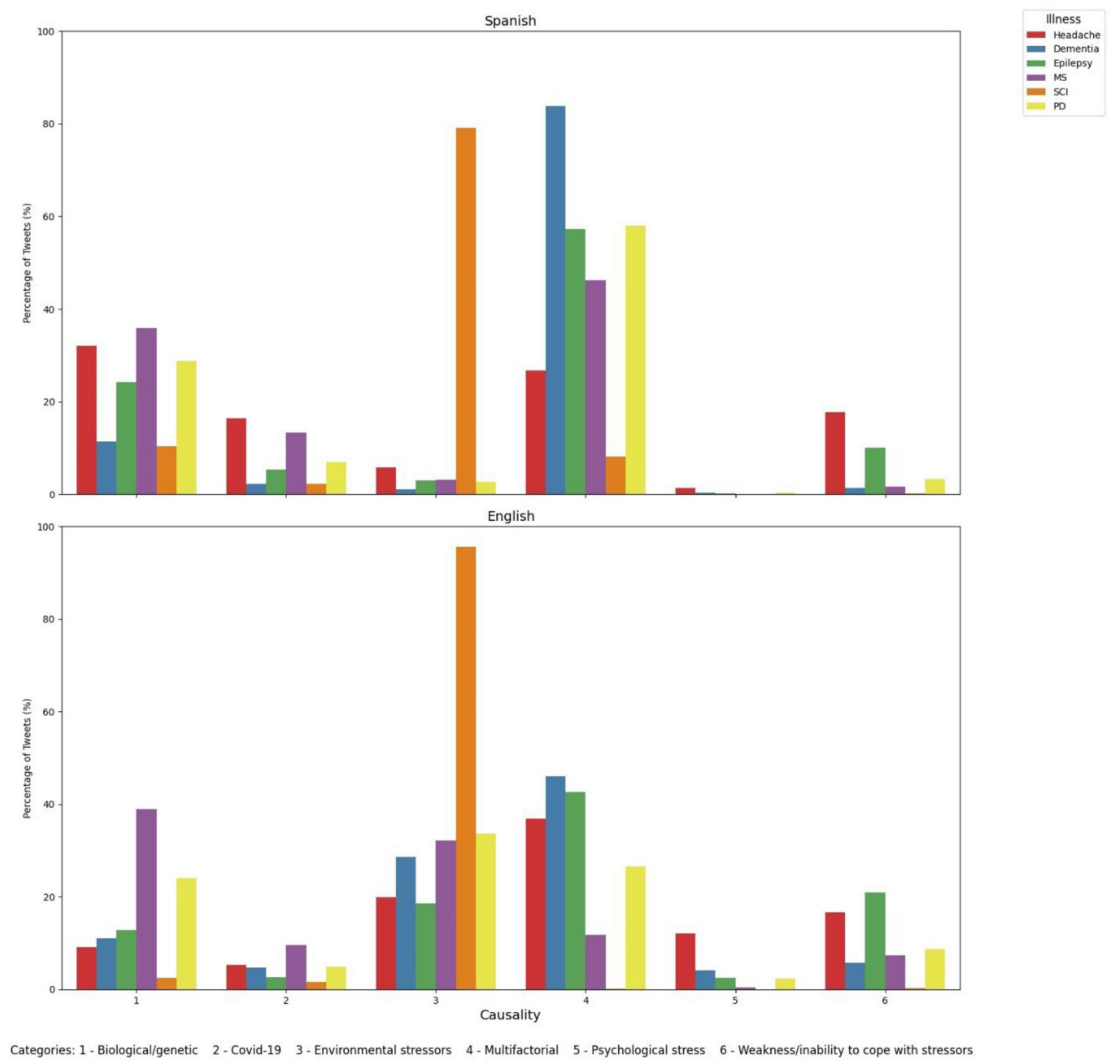
Social perception around causality of dementia and other neurological disorders in English and Spanish tweets. MS, Multiple sclerosis; SCI, spinal cord injury; PD, Parkinson's disease.

Another objective of our study was to understand the public perception around the causality of dementia and other neurological disorders, collected in Figure 7. We observed that both English and Spanish tweets perceived dementia as the most common etiology related (46.02% and 83.78%, respectively). However, they differed in terms of the second most common cause, with English tweets highlighting environmental stressors (28.53% vs. 1.03% in Spanish Tweets). For Spanish tweets, biological/genetic causes represent the second causative agent of dementias (11.4% vs. 10.98% in English tweets). We can also observe that English tweets specifically highlight different etiological agents related to dementia when compared to Spanish tweets, including COVID-19 (4.67% vs. 2.2%), difficulties to face stressors (5.66% vs. 1.29%) and psychological stressors (4.15% vs. 0.31%). When compared to other neurological disorders, dementia ranks first in both English and Spanish tweets for the category “multifactorial”, MS ranks first as biological/genetic causes for both languages, and in COVID-19 for English tweets, representing the second after headache in Spanish tweets. Epilepsy and headache are two predominant conditions perceived as difficulties to face stressors, SCI as a condition related to environmental stressors and headache with psychological stressors.

### 3.5 Comparative analysis of non-medical tweets reveals varied perspectives on dementia and neurological disorders in English and Spanish tweets

**Figure 8:**
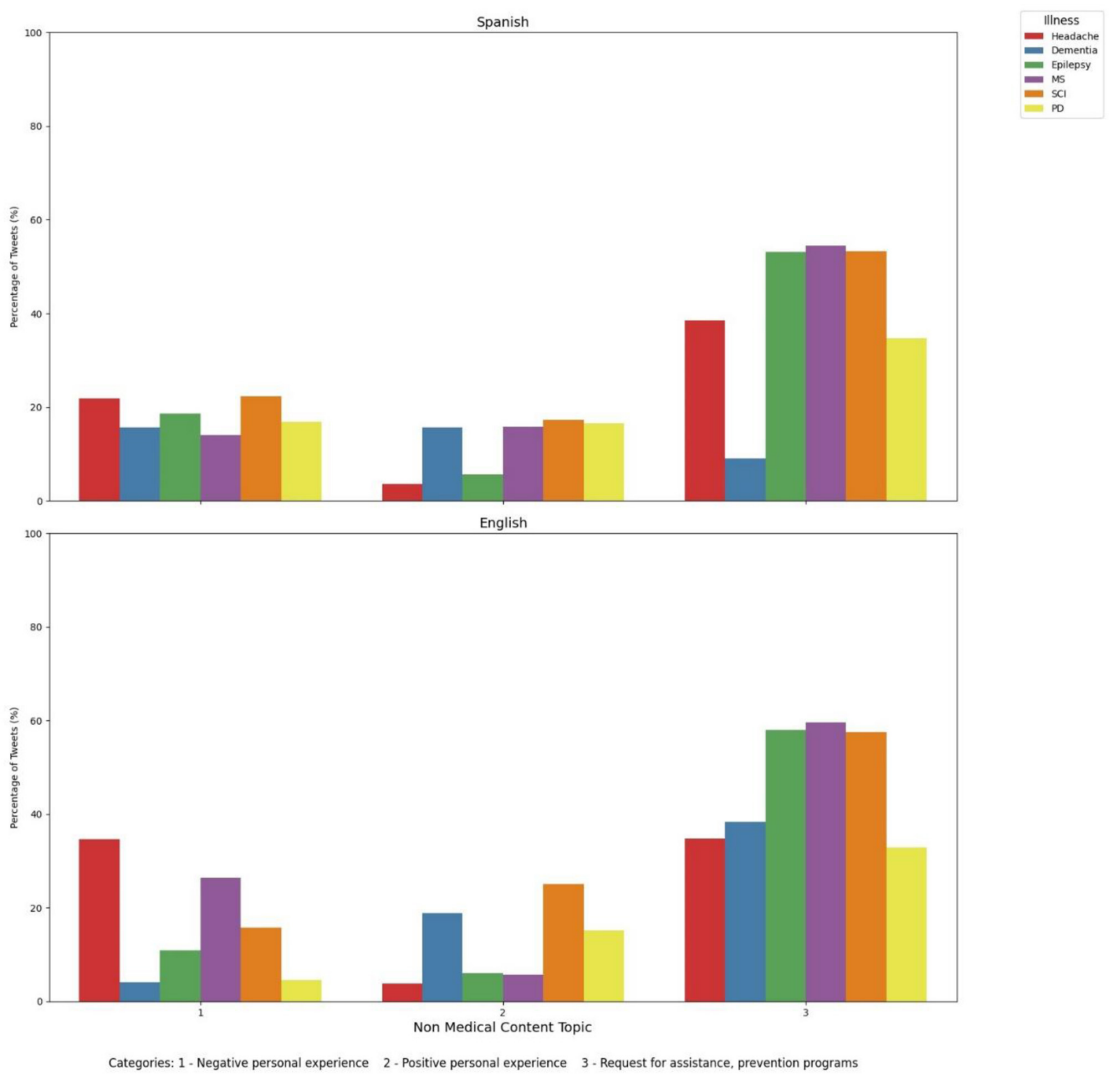
Non-medical content of tweets discussing dementia and other neurological disorders in English and Spanish tweets. MS, Multiple sclerosis; SCI, spinal cord injury; PD, Parkinson's disease.

Lastly, non-medical content related to dementia and other neurological disorders were analyzed (Figure 8). We can observe that prevention programs/petition of help are the most common topic for English tweets (38.32%), whereas for Spanish tweets it only represents 8.96%. We can also highlight that positive personal experiences are similarly reported in English and Spanish tweets (18.91% and 15.65%), whereas negative personal experiences are notably higher in Spanish tweets when compared to English tweets (15.66% vs. 4.11%). It was also remarkable for us that, compared to other neurological disorders, dementia was the last entity found in Spanish tweets and the fourth in English tweets. MS, SCI and epilepsy were the three disorders with the greatest petitions of prevention programs and help (59.54%, 57.5% and 57.94%, respectively for English tweets and 54.48%, 53.21% and 53.06%, respectively for Spanish tweets). Headache was the first neurological disorder associated with negative personal experiences for English tweets and the second for Spanish tweets, closely followed by SCI. Also, SCI was the neurological disorder more commonly associated with positive personal experiences for both languages, closely followed by dementia, and also by MS and PD in Spanish tweets.

## 4 Discussion

In the present work we have evaluated the relevance of X as a social platform to discuss various critical concerns related to dementia. Including English and Spanish Tweets and facing dementia to other neurological disorders, our results have found that dementia is, by far, the most common neurological disorder commented in X. Likewise, we show that dementia is the first neurological disorder trivialized in Spanish Tweets and the second in English Tweets included in our study, with some important differences reported in the geolocation data. In parallel, we also observe significant differences in the perception of dementia treatment between Spanish and English Tweets, as well as in their associated sentiments. Eventually, differential perceptions around medical content (etiology) and non-medical content (positive/negative experiences and aid petitions) in dementia and other neurological disorders have also been detected in our study, revealing the complex landscape associated with dementia and neurological disorders in the Twittersphere.

First, it is remarkable for us that dementia ranks by far as the most commented neurological disorder in X. The huge impact of dementia in our society is undeniable. Currently, the medical management of dementia has an associated cost of USD 600 billion globally, with 47 million people in the world with dementias, although this number is expected to increase almost three times, up to 131 million, by 2050 (2). Epidemiological data shows that dementia represents one of the major causes of burden related to neurological disorders globally (33). However, we were surprised by the large discrepancy in the number of tweets about dementia compared to the other neurological diseases included in our study, which all have a big impact on society. For instance, despite dementia is found to be included in the top 25 causes of years lived with disability (YLDs), migraine and epilepsy are ahead of dementia according to available literature (34). Epidemiological data have rated headache disorders (particularly migraines) as the second leading cause of disability after back pain in terms of years lived with disability, accounting for 3% of all visits to emergency services each year (35). Thus, the large discrepancy in the number of tweets concerning dementias and headaches or epilepsy is surprising, given that previous research on X has claimed the platform's usefulness in headache condition conversations (36). Besides, the literature also remarks the significance in terms of prevalence and impact of the remaining neurological disorders collected in our study like MS, PD and SCI (37).

The poor representation in tweets of disease entities like MS and SCI comparing to dementia is particularly surprising, given their severe psychosocial consequences for patients, relatives, healthcare professionals, and the society (38, 39). Despite of this fact, previous works have also demonstrated the relevance of X as an useful tool to understand public perception of both MS and SCI (40–42). Thus, our findings demonstrate significant inequity on X in terms of the consideration of various neurological disorders, with dementias receiving the most attention while other entities, such as SCI and MS, being noticeably neglected in comparison. A higher presence of users, specialists, or platforms dedicated to these underrepresented illnesses may benefit afflicted patients or family members who use this social network for support or other objectives.

Besides, we observed a maximum peak in 2013 for Spanish Tweets and in 2020 for English Tweets, which suggests that there may be cultural and socioeconomic influences that affect the way people discuss certain topics in different regions and at different times. We have not been able to find a specific event relating this peak in 2013 with dementia in Spanish tweets; however, it could be attributed to several factors that elevated public discourse around neurodegenerative diseases. One major contributor was the aging population in Spain and Latin America, which brought increasing awareness of Alzheimer's disease and other forms of dementia. As concerns grew about the long-term care and health implications for elderly populations, campaigns such as World Alzheimer's Day and organizations like the Spanish Confederation of Alzheimer's (CEAFA) played a pivotal role in launching initiatives aimed at educating the public about dementia. The growing public dialogue likely coincided with a rising adoption of social media in Spanish-speaking regions, allowing for broader engagement on these pressing issues. These combined factors likely led to the noticeable increase in discussions about dementia on X during this period.

The peak in 2020 with English tweets coincided just with the start of the COVID-19 pandemic. In a systematic review including 22 studies evaluating the impact of COVID-19 in dementia comprised between 2020 and 2021, Gaigher et al. (43) reported that social isolation and lockdown leaded to the emergence or increase of neuropsychiatric symptoms and motor difficulties, with some heterogeneous findings concerning the pandemic's impact on the cognition of people with dementia. They also found that caregivers suffered from the pandemic's impact, experiencing an increase in the burden of care and symptoms of stress, depression, and anxiety. Besides, the relatives of the affected patients also showed a significant impact during this period (44). Therefore, and in agreement with previous results (19), our study indicates that X was a platform used to reflect this social context. However, and once again, it is still surprising for us not to observe these changes in other neurological entities and also the differences reported in Spanish Tweets, evidencing the complexity of social dynamics and their reflection on platforms and social networks such as X. Additionally, research emerging in 2020 began to investigate potential long-term neurological effects of COVID-19, including cognitive decline and dementia-like symptoms, further fueling discussions (45). Another key factor was the anticipation and debate surrounding the approval of new treatments for Alzheimer's disease, such as aducanumab, which was under regulatory review in that period (46). In this line, past works conducted in Twitter observed a significant number of tweets posted discussing the recent Food and Drug Administration's approval of this drug in 2021 (47), therefore defining the impact of novel drugs used in the management of dementias in this platform.

Secondly, we observed that dementia was the first neurological disorder trivialized in Spanish tweets and the second in English tweets, whereas headache followed just the opposite pattern. The relationship between dementia and social stigma/trivialization in X has been noticed in past works. Authors like Bacsu et al. (17) attributed the content of tweets discussing social stigma/trivialization of dementia to four major themes: (1) ageism and devaluing the lives of people with dementia, (2) disinformation and erroneous beliefs about dementia and COVID-19, (3) dementia being used as an insult for political derision, and (4) addressing the stigma around dementia. It is remarkable for us as well that more than 60% of Spanish tweets trivialized dementia, whereas headache and dementia did not exceed 40% of analyzed tweets in English. The percentage of tweets trivializing dementia described found in previous studies were notably smaller in comparison to our results (14, 48), although Cheng et al. (13) found that approximately 40% of English tweets (*n* = 398 tweets) written between January and February in 2018 treating dementia were related to social stigma. Our study has included a broad number of tweets comprised between 2009 to 2023, also considering regional differences between English and Spanish Tweets. Our study may reflect sociocultural differences when using the term “dementia” between Spanish-speaking and English-speaking cultures. On the other hand, past works have found that English and Spanish tweets of dementia caregivers present significant differences in their content, demonstrating the relevance of including sociocultural variables for evaluating different topics (49). To our best knowledge no studies have specifically focused on the reasons underlying the differences between Spanish and English tweets in terms of dementia trivialization. Therefore, this could be an interesting topic of research in X for future works. Likewise, despite we have not deepened on the temporal evolution of tweets trivializing dementia in English and Spanish tweets, future works could also be aimed to describe possible differences in patterns of dementia misuse. Geolocation data show a greater number of tweets trivializing dementia in countries like United States or Spain. However, it must also be highlighted that trivialization of dementia is observed in a wide spectrum of nations and languages and represents a global, rather than a country or region-specific concern (13, 14). We totally agree with previous works concluding that notwithstanding social media has been used to perpetuate stigma, it may also be used to disprove negative views, stereotypes, and misinformation, leading to a better use of this platform (17).

Thirdly, dementia is considered by Spanish and English Tweets mostly an incurable disease, and little tweets considered them treatable with professional aid. Besides, our sentiment analysis data show that this fact is associated with negative feelings like fear and sadness and other sentiments in English Tweets whereas other and joy feelings were more clearly related to Spanish tweets. Dementia is a terminal and incurable illness, not always recognized as such, that represents the 6th leading cause of death in the United States and is one of the few leading causes of death where the age-adjusted death rate is increasing (50). Thus, it is surprising that some tweets considered dementia as treatable with professional help or self-manageable, particularly in English tweets. Healthcare professionals can provide palliative cure directed to the psychosocial, physical, emotional, and spiritual symptoms, but cannot treat dementia despite their obvious efforts and remarkable work (51). In the new era of dementia's management, treatment with monoclonal antibodies to modulate disease progression and cognitive decline, the search of potential biomarkers with translational applications and early detection in preclinical or prodromal stages will be crucial for future clinical care (52).

Moreover, it is critical to remark that despite dementias are not curable they are preventable, and a special focus on this message should be made in this regard (53). Promising dementia prevention strategies include controlling vascular risk factors, engaging in cognitive, social, and physical activities, maintaining a healthy diet and addressing depression and psychosocial stress (54, 55). Therefore, a greater emphasis on the prevention of dementia should be done, and X could be an excellent platform to achieve this objective. Indeed, past works have claimed the significant impact of X on neurology, enabling education, research promotion, and global outreach (11). While it enhances patient support and research communication, caution is advised due to potential misinformation, such as it might be occurring herein, and in some cases to unprofessional conduct, emphasizing the importance of establishing ethical guidelines and broader efforts to communicate verifiable information in this platform. On the other hand, and despite not being a central objective of the study, some surprising results might also be highlighted when compared disease perceptions between English with Spanish tweets. For instance, more English tweets perceive SCI as treatable with professional aid whereas for Spanish tweets almost the same percentage of tweets conceive this condition as either treatable with professional aid or incurable. Future works could deepen on the underlying explanations of these discrepancies.

The distinctive emotional responses observed in English and Spanish tweets regarding neurological disorders likely reflect cultural differences in attitudes toward mental health and disease management. In English-language tweets, perceptions of neurological disorders as non-curable evoke a spectrum of intense emotions such as fear, sadness, and, to a lesser extent, joy and anger. This probably suggests a high level of emotional engagement and concern, potentially influenced by greater public awareness and discourse around mental health in English-speaking communities. Conversely, Spanish-language tweets demonstrate a more varied emotional landscape, with joy and other sentiments being more prominent and fear being less represented. This might indicate a different cultural approach to discussing neurological disorders, possibly shaped by varying levels of stigma, public education, or healthcare infrastructure. The relative absence of fear and sadness in Spanish tweets could reflect a more optimistic or less anxious societal view of these conditions, while the focus on other sentiments might highlight a different set of values or coping mechanisms in the Spanish-speaking world.

On the other hand, dementia is mostly considered as a multifactorial disease by English and Spanish tweets, but in case of the former, the second most common cause of dementia discussed in this platform is attributed to environmental stressors, whereas for Spanish tweets is more regarded as biologic/genetic mechanisms. Overall, pathophysiological signatures of dementias are broadly characterized by the aggregation of misfolded proteins (such as amyloid-β plaques and neurofibrillary tangles in Alzheimer's disease) and cerebrovascular disease (56). Scientific literature agrees that dementias are the result of both genetic and environmental factors, which may drive to different etiological factors responsible for the initiation and development of dementia (57, 58). English tweets seem to give a broader relevance to environmental factors and to weakness/inability to deal with these factors in comparison to the tweets written in Spanish. It should be noticed here that some studies give to heritability a weight of up to 60–80% in case of dementia like Alzheimer's disease, identifying a set of genetic loci potentially involved in the risk of this disease (59). Therefore, a broader understanding and dissemination on the role currently accepted of genetic and environmental factors in X should be carefully made, particularly for those defending the weakness/inability to deal with stress as a etiological agent of dementia.

Likewise, a significant number of English and to a lesser extent, Spanish tweets points to the virological agent of the COVID-19 -SARS-CoV-2- as a causative mechanism of dementia. Scientific literature seems to support this, evidencing that SARS-CoV-2 has neuroinvasive and neurotropic characteristics with acute and chronic neurovirulent potential; and together with its ability to induce myocardial and systemic vascular damage along with thrombosis and multiorgan damage it is suggested that each injury consequence has the independent potential to contribute to long-term cognitive deficits with the possibility of progressing to or worsening pre-existing dementia (60). Thus, our results show that the role of SARS-CoV-2 in dementia might have been a common topic in X. Besides, psychological/mental factors like depression, social isolation, stress, anxiety, sleep quantity/quality and loneliness are important risk factors related to dementia (61). We have also identified differences in the number of tweets considering these factors between English- and Spanish-speaking cultures, which may indicate that this critical factor related to dementia might be obviated in case of the latter.

Finally, our results also report that petitions of aid and prevention programs are the most common topics of tweets with no medical content treating dementia but for Spanish tweets, positive and negative personal experiences of dementia were more common. Likewise, we can observe how MS, epilepsy and SCI are the three neurological disorders with greater petitions of aids, with dementia representing the fourth neurological disease ranked for English tweets and the last for Spanish tweets. One possible hypothesis for our observations could be rooted in cultural factors and societal perceptions surrounding dementia within Spanish-speaking communities. As we highlighted before, a significant number of Spanish tweets trivialized dementia, which may explain the lack of representation of tweets seeking help.

On the other hand, the use of X for commenting positive and negative experiences related to dementia has been documented previously (62, 63). We observed that a similar proportion of personal and negative experiences can be reported in Spanish Tweets, whereas for English tweets a broader number of tweets relating positive experiences can be reported. Spanish-speaking cultures may lean toward openly expressing problems, resulting in a higher proportion of negative experiences shared. In contrast, English-speaking cultures may emphasize positivity, leading to a broader range of tweets reflecting positive experiences, even in challenging contexts like discussing dementia. These differences could also be influenced by the stigma; however broader efforts should be placed in this sense. Likewise, it was remarkable for us the different representation seeking for help in the different various neurological diseases. The visibility and acute nature of symptoms associated with conditions like MS and epilepsy may prompt individuals to seek help and share their experiences more actively on social media. Additionally, advocacy efforts and awareness campaigns surrounding these entities may contribute to a higher volume of tweets seeking support and information. Howsoever, past works have evidenced that X is a platform in which people affected with dementia, their caregivers, relatives and health institutions also claim for help and broader efforts in prevention and awareness programs (16, 64, 65). Further studies should place attention on the use of X to seeking help in the context of neurological disorders, particularly for the previously mentioned diseases.

### 4.1 Limitations

One limitation of our study is the non-representativeness of tweets in capturing broader societal attitudes toward dementia and neurological disorders. This arises because discussions on the studied topics may not include the perspectives of individuals who do not use X or who do not actively engage in mental health conversations on the platform. Additionally, X's character limit constraints may oversimplify or lack nuance in addressing complex topics, potentially resulting in a superficial understanding of user sentiments. Finally, it is possible that tweets using contractions or slang to describe neurological disorders could have been unnoticed by this work. Future research could enhance these findings by incorporating data from other social media platforms and employing alternative data collection methods, such as in-depth interviews.

## 5 Conclusions

The current study delves into the significance of X as a social platform for addressing diverse critical issues concerning dementia, comparing this condition with other neurological disorders in English and Spanish Tweets. Our findings underscore dementia as the predominant neurological disorder discussed on X, with an important number of tweets trivializing this condition, especially in Spanish tweets. Moreover, discernible variations in dementia treatment perceptions and associated sentiments emerge between Spanish and English Tweets. Furthermore, our analysis unveils contrasting perspectives on medical (causality) and non-medical aspects of dementia and other neurological disorders, illuminating the intricate landscape within the Twittersphere surrounding these conditions. Future works could be conducted to continue deepening on the relevant use of this social media in the understanding of public perceptions and necessities of X users in dementia and neurologic disorders.

## Data Availability

The raw data supporting the conclusions of this article will be made available by the authors, without undue reservation.
